# NF-κB, p38 MAPK, ERK1/2, mTOR, STAT3 and increased glycolysis regulate stability of paricalcitol/dexamethasone-generated tolerogenic dendritic cells in the inflammatory environment

**DOI:** 10.18632/oncotarget.4234

**Published:** 2015-05-22

**Authors:** Klára Dáňová, Anna Klapetková, Jana Kayserová, Anna Šedivá, Radek Špíšek, Lenka Palová Jelínková

**Affiliations:** ^1^ Sotio a.c., Prague, Czech Republic; ^2^ Department of Immunology, Charles University, 2nd Medical School, Prague, Czech Republic

**Keywords:** tolerogenic DCs, immunoregulation, stability, activation pathways, glycolysis

## Abstract

Tolerogenic dendritic cells (tDCs) may offer an intervention therapy in autoimmune diseases or transplantation. Stable immaturity and tolerogenic function of tDCs after encountering inflammatory environment are prerequisite for positive outcome of immunotherapy. However, the signaling pathways regulating their stable tolerogenic properties are largely unknown. In this study, we demonstrated that human monocyte-derived tDCs established by using paricalcitol (analogue of vitamin D2), dexamethasone and monophosphoryl lipid A exposed for 24h to LPS, cytokine cocktail, polyI:C or CD40L preserved reduced expression of co-stimulatory molecules, increased levels of inhibitory molecules ILT-3, PDL-1 and TIM-3, increased TLR-2, increased secretion of IL-10 and TGF-β, reduced IL-12 and TNF-α secretion and reduced T cell stimulatory capacity. tDCs further induced IL-10-producing T regulatory cells that suppressed the proliferation of responder T cells. In the inflammatory environment, tDCs maintained up-regulated indoleamine 2, 3 dioxygenase but abrogated IκB-α phosphorylation and reduced transcriptional activity of p65/RelA, RelB and c-Rel NF-κB subunits except p50. Mechanistically, p38 MAPK, ERK1/2, mTOR, STAT3 and mTOR-dependent glycolysis regulated expression of ILT-3, PDL-1 and CD86, secretion of IL-10 and T cell stimulatory capacity of tDCs in the inflammatory environment. Stability of tDCs in the inflammatory environment is thus regulated by multiple signaling pathways.

## INTRODUCTION

Dendritic cells (DCs) are specialized antigen presenting cells that, depending on their activation status, can induce tolerance or immunity [1]. Tolerogenic DCs (tDCs) can be generated from precursor cells *in vitro* and represent potentially promising tool for inducing or restoring immune tolerance in the context of transplantation and autoimmune diseases [2]. Tolerogenic DCs are usually defined by low or intermediate expression of co-stimulatory molecules CD80, CD86 and CD40 in contrast to high levels of inhibitory factors such as immunoglobulin-like transcript (ILT) molecules 2, 3, 4, and/or programmed death ligand (PDL)-molecules. Additionally, tDCs secrete low amounts of pro-inflammatory cytokines and high quantities of anti-inflammatory cytokines, such IL-10. This results in the attenuation of T cell stimulatory capacity and/or induction and expansion of T regulatory cells (Tregs). Different approaches targeting DCs differentiation and function have been shown to establish tDCs *in vitro* [3-5]. Notably, dexamethasone (Dex) and/or vitamin D (VitD) receptor agonists (1,25(OH)_2_D3 and its analogues) have been described to generate tDCs through the suppression of NF-κB-dependent DCs maturation [6, 7]. Such Dex/VitD conditioned tDCs have been shown to acquire a robust immunoregulatory phenotype and are currently tested in early stage clinical trial in patients with rheumatoid arthritis [8].

One of the major concerns associated with *in vivo* administration of *in vitro* established tDCs is their functional stability. Once injected into patients with chronic inflammation, such as autoimmune disease, tDCs should remain stable and retain their tolerogenic properties in the absence of tolerogenic agents. Furthermore, there is a potential risk that *ex vivo* differentiated tDC might switch to an activated phenotype when encountering pro-inflammatory signals *in vivo* and contribute to the further expansion of the autoimmune reaction.

The pro-inflammatory DC maturation initiated by pathogen associated molecular patterns or by inflammatory cytokines is connected with the activation of numerous signaling pathways including transcription factor NF-κB and p38 mitogen-activated protein kinase (MAPK) pathway [9, 10]. Recently, the mammalian target of rapamycin (mTOR) signaling pathway has been reported to coordinate the production of pro- versus anti-inflammatory cytokines in human monocytes and DCs through regulating NF-κB and signal transducer and activator of transcription 3 (STAT3) activity [11, 12]. The pattern of activated signaling events triggered in tolerogenic DC maturation is profoundly different and is associated namely with activation of extracellular-signal-regulated kinase (ERK) 1/2 and non-canonical NF-κB pathway [13-15]. However, little is known about the signaling pathways triggered in tDCs after encountering inflammatory environment and their role in preserving tolerogenic properties of tDCs.

In this study, we established a good manufacturing practice (GMP)-compliant protocol for the human tDCs differentiation using paricalcitol (19-nor-1, 25-dihydroxyvitamin D2), synthetic analogue of active form of VitD2 that retains significant immunomodulatory activity [16] and immunosuppressive drug dexamethasone (Dex). Finally, VitD2/Dex-generated tDCs (Dex/VitD2 tDCs) were activated with monophosphoryl-lipid A (MPLA), a non-toxic alternative of lipopolysaccharide (LPS), to obtain so-called “alternatively activated tDCs” with improved tolerogenic properties as reported previously [17]. We comprehensively tested their phenotypic and functional stability after mimicking inflammatory environment by using LPS, cocktail of pro-inflammatory cytokines (CC), polyinosinic:polycytidylic acid (polyI:C) and CD40L. In our study, we addressed for the first time a detailed analysis of molecular mechanisms responsible for the maintenance of stable tolerogenic properties of tDCs in the inflammatory environment.

## RESULTS

### tDCs preserved semimature tolerogenic phenotype after restimulation with LPS, CC, polyI:C and CD40L

To study the functional properties and stability of tDCs, we cultured freshly isolated human monocytes in GMP–compliant medium Cell Gro in the presence of GM-CSF, IL-4, and tolerogenic factors Dex and VitD2. Control DCs (cDCs) were cultured without Dex and VitD2. Finally, DCs were activated with MPLA.

**Figure 1:**
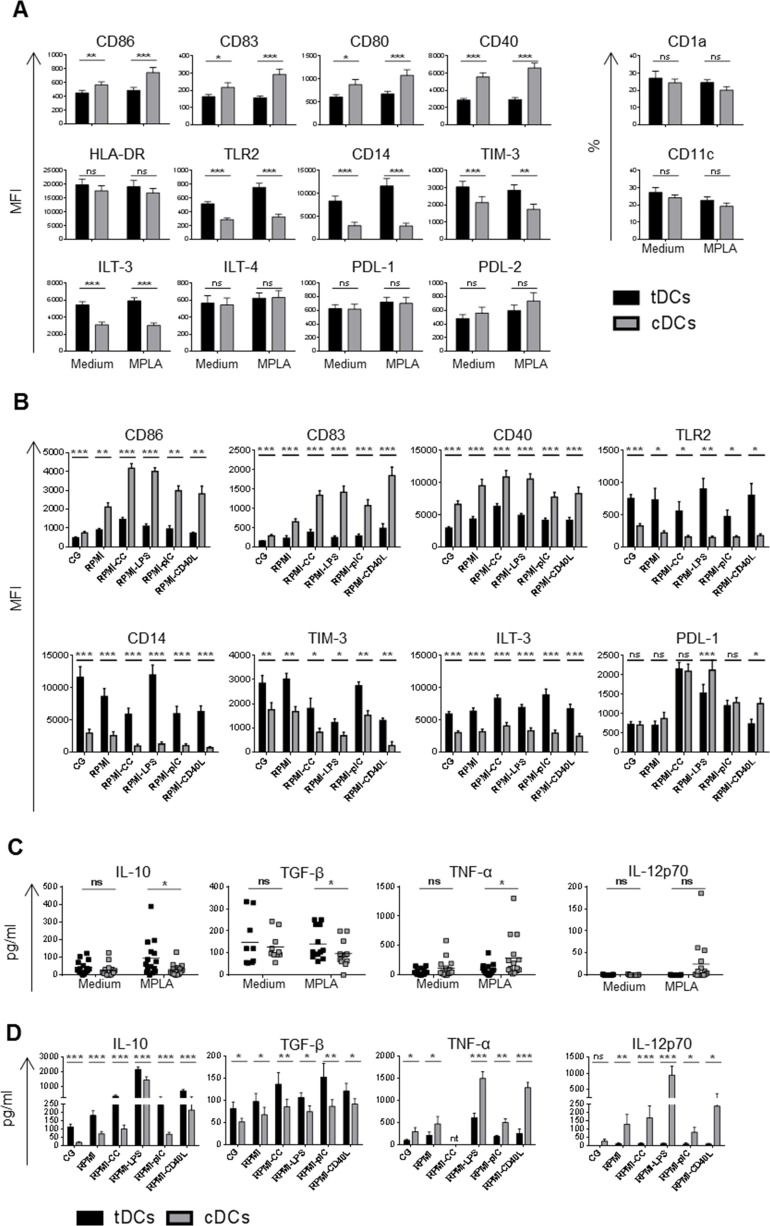
Dex/VitD2 tDCs exhibit a stable semimature phenotype and anti-inflammatory cytokine secretion profile DCs were differentiated from monocytes in Cell Gro supplemented with GM-CSF and IL-4 in presence (tDCs, black bars) or absence of Dex and VitD2 (cDCs, grey bars) to obtain immature tDCs or immature cDCs (MEDIUM). Cells were finally activated with MPLA for 24 h (MPLA). **A.** Surface marker expression was analyzed by flow cytometry and **C.** cytokines released by DCs were analyzed from supernatants by Luminex (tDCs black squares, cDCs grey squares). After activation with MPLA for 24 h in Cell Gro (CG), cells were washed and recultured in complete RPMI without tolerising factors and treated with cytokine cocktail (CC) containing IL-1β, TNF-α, IL-6 (all 10 ng/ml) and IFN-γ (100 ng/ml) or LPS (1 μg/ml) or polyI:C (25 μg/ml) or CD40L (1000 ng/ml) or they were left unstimulated (RPMI). **B.** Bar graphs represent surface marker expression analyzed by flow cytometry and **D.** cytokines released by DCs analyzed by Luminex or ELISA after 24 h of restimulation. Data represent MFI ± SEM or percentages of positive cells (CD1a and CD11c expression) from at least 3 independent experiment and minimal 10 donors. *p ≤ 0.05, **p ≤ 0.01, ***p ≤ 0.001 (paired *t*-test), nt-not tested.

As shown in Figure 1A and Supplementary Figure 1, tDCs cultured in Cell Gro exhibited tolerogenic phenotype with significantly lower surface levels of CD86, CD83, CD80 and CD40 but higher levels of Toll-like receptor (TLR)-2, CD14 and inhibitory molecules TIM-3 and ILT-3 in comparison to cDCs. The levels of CD1a, CD11c, HLA-DR and inhibitory molecules ILT-4, PDL-1 and PDL-2 were comparable in tDCs and cDCs. To study the stability of DCs, cDCs and tDCs generated in Cell Gro were recultured in complete RPMI without tolerising agents and subsequently stimulated with LPS, CC, polyI:C and CD40L for 24 h (Figure 1B). Restimulation led to a slight upregulation of CD86, CD83 and CD40 on tDCs, however, it remained significantly lower when compared to cDCs. Importantly, the expression of TLR2, CD14 and ILT-3 on tDCs remained high after restimulation when compared to cDCs. The expression of TIM-3 decreased approximately two-fold after CC, LPS and CD40L stimulation, however, it remained higher in comparison to cDCs. The expression of tolerogenic molecule PDL-1, that was low on tDCs from Cell Gro, dramatically increased after restimulation of tDCs with CC as well as LPS and slightly after polyI:C stimulation for 24 h.

In line with tolerogenic cell-surface phenotype, tDCs produced higher levels of IL-10 and TGF-β, low quantities of TNF-α and no IL-12p70 as compared to cDCs (Figure 1C). Subsequent restimulation of tDCs with CC, LPS, polyI:C or CD40L led to robust increase of IL-10 production, slight up-regulation of TGF-β, low production of TNF-α and minimal IL-12 production. (Figure 1D). Collectively, these data demonstrate that, in spite of the presence of maturation stimuli, Dex/VitD2 tDCs preserve non-proinflammatory profile with high expression of tolerogenic markers, high IL-10/IL-12p70 ratio and sustained TGF-β production.

### Dex/VitD2 tDCs preserved reduced T cell stimulatory capacity after restimulation

**Figure 2:**
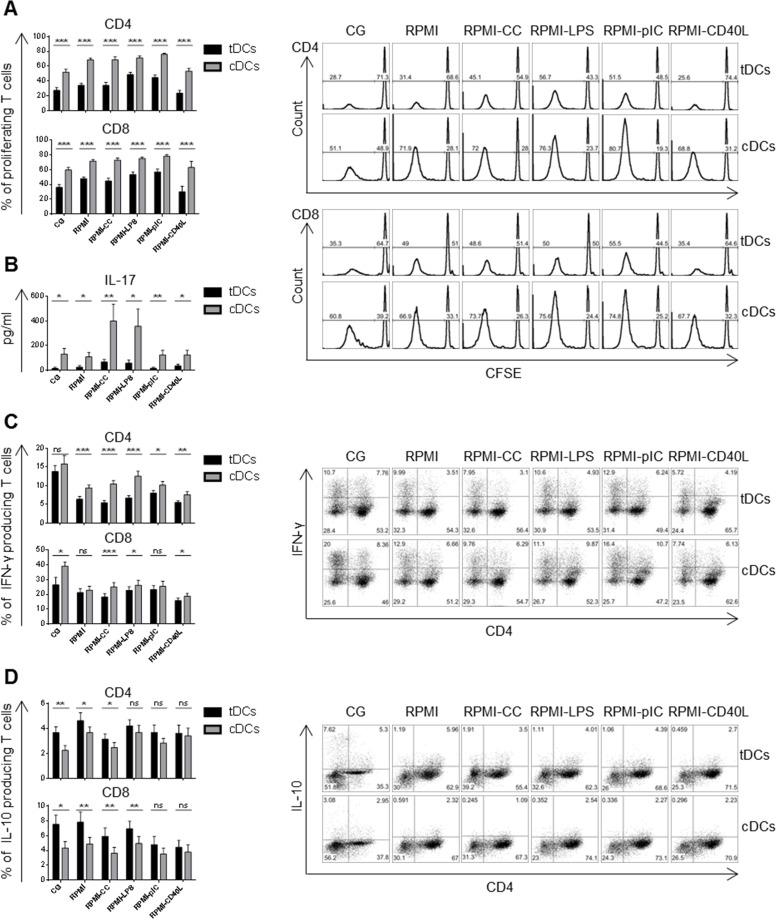
Dex/VitD tDCs maintain reduced T cell stimulatory capacity after restimulation DCs were differentiated in Cell Gro in presence (tDCs) or absence of Dex and VitD2 (cDCs) and activated with MPLA (CG). Then, DCs were washed, recultured in complete RPMI without tolerising factors and treated with cytokine cocktail (CC) described in Figure 1B or LPS (1 μg/ml) or polyI:C (25 μg/ml) or CD40L (1000 ng/ml) or they were left unstimulated (RPMI). After 24 h, tDCs and cDCs were washed and incubated with allogeneic T cells at 1:10 ratio (DCs/T cells). **A.** Proliferation of T cells was assessed on day 6 by CFSE dilution method. Percentages of proliferating T cells and representative histograms are shown. **B.** Production of IL-17A in DCs/T cell co-cultures was analyzed by ELISA on day 6. **C.** Percentages of IFN-γ producing T and **D.** IL-10 producing T cells was assessed on day 6 or day 9, respectively. Representative dot plots are shown. Data represent mean ± SEM for at least 3 independent experiments and at least 10 donors. *p ≤ 0.05, **p ≤ 0.01, ***p ≤ 0.001 (paired *t*-test).

TDCs or cDCs were cultured with allogeneic T cells at a ratio of 1:10. TDCs were weaker inducers of CD4+ as well as CD8+ T cell proliferation, even after the restimulation, irrespective of the maturation agent when compared to cDCs (Figure 2A). In line with this, tDCs induced low IL-17A production by allogeneic T cells even after restimulation in contrast to cDCs that were potent inducers of IL-17A by T cells especially after CC and LPS stimulation (Figure 2B). Moreover, co-incubation of allogeneic T cells with tDCs cultured in Cell Gro skewed the T cell cytokine profile towards reduced IFN-γ and significantly increased IL-10 production by CD4+ as well as CD8+ T cells, in comparison to cDCs (Figure 2C, 2D). In addition, co-incubation of T cells with tDCs restimulated with CC, LPS, polyI:C and CD40L led to marked reduction of CD4+ IFN-γ producing T cells together with stable numbers of CD4+ IL-10 producing T cells. The percentage of CD8+ IFN-γ producing T cells remained stable or slightly decreased after CC and CD40L restimulation of tDCs, while the amount of CD8+ IL-10 producing T cells remained almost the same after restimulation of tDCs with LPS and slightly decreased after restimulation of tDCs with CC, polyI:C and CD40L.

### Dex/VitD2 tDCs induced Tregs differentiation from naïve CD4+ T cells

**Figure 3:**
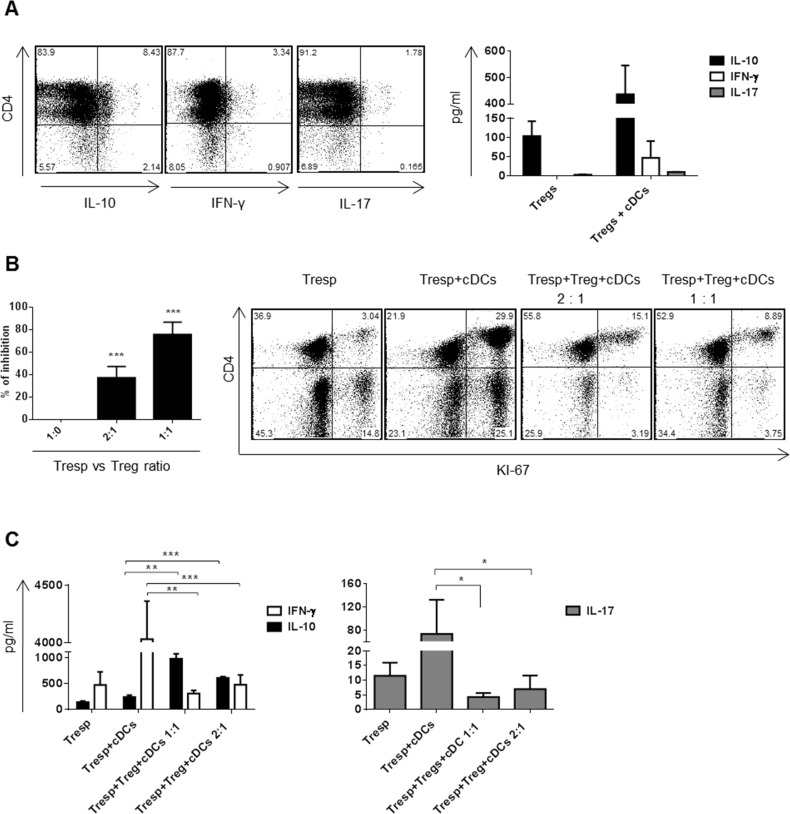
Dex/VitD2 tDCs induce IL-10 producing Tregs that are able to suppress proliferation of responder T cells DCs (donor B) were differentiated in Cell Gro in presence (tDCs) or absence (cDCs) of Dex and VitD2 and activated with MPLA. Dex/VitD2 tDCs were incubated with allogeneic T cells (donor A) at 1:10 ratio (DCs/T cells) in RPMI (5% human AB serum) for two rounds of priming. Then, the cytokine production and suppressive capacity of induced Tregs was evaluated. **A.** Tregs (donor A) were co-cultured with specific cDCs (donor B) at 1:10 ratio (DCs/T cells). Representative dot plots from 3 independent donors show percentages of IL-10, IFN-γ and IL-17 producing T cells assessed on day 6. Production of IL-10, IFN-γ and IL-17 was analyzed in cell supernatants by ELISA on day 6. **B.** CD4+ Tregs were tested for suppressive capacity in MLR assay. CD4+ Tregs (donor A) were plated with responder T cells (donor A) and cDCs (donor B). cDCs were from the same donor as the Dex/VitD2 tDCs used to induce Tregs. Cells were plated in a Treg/Tresp/DCs ratio of 10:10:1 or 5:10:1. As additional controls, Tresp were cultured alone or with cDCs. After 6 d, cells were recovered and proliferation of responder cells was analyzed by measuring KI-67 by flow cytometry. The percent inhibition of responder T cell proliferation (black bars, mean ± SEM for 3 independent donors, each performed in triplicate) and one representative dot plot showing proliferation of responder T cells are depicted. **C.** Cell culture supernatants were recovered for IL-10, IFN-γ and IL-17A analysis. Data represent mean ± SEM for 3 independent donors (each performed in triplicate). *p ≤ 0.05, **p ≤ 0.01 (paired *t*-test).

Increased capacity to promote differentiation/induction of Tregs from naïve precursors seems to be one of the most important hallmarks of tDCs [19]. We showed that co-culturing of allogeneic T cells with Dex/VitD2 tDCs induces higher and stable levels of IL-10 producing CD4+ T cells when compared to cDCs. Previously, IL-10 producing CD4+ T cells generated by repetitive priming of CD4+ naïve T cells with immature DCs or tDCs generated by VitD3 were shown to display regulatory properties [20, 21]. Thus, to test whether IL-10 producing CD4+ T cells induced by Dex/VitD2 tDCs (referred to as Tregs) possess regulatory activity after being expanded by repetitive priming, we cultured naïve CD4+ T cells with allogeneic Dex/VitD2 tDCs for two rounds of stimulation. As shown in Figure 3A, Tregs expanded by Dex/VitD2 tDCs produced IL-10 but virtually no IFN-γ and IL-17A. IL-10 production was increased upon specific activation with cDCs. IFN-γ and IL-17 production was only slightly increased upon specific activation with cDCs. To analyze the suppressive function of Tregs expanded after two rounds of stimulation with Dex/VitD2 tDCs, Tregs were titrated into a MLR comprising allogeneic cDCs (from the same DCs donor as used in the original stimulation) and autologous responder T cells (from the same T cell donor as Tregs). As shown in Figure 3B, Tregs dose-dependently inhibited responder T cell proliferation. Moreover, adding of Tregs into MLR led to up-regulation of IL-10 and down-regulation of IFN-γ and IL-17A production in a dose-dependent manner (Figure 3C). Therefore, IL-10 producing Tregs induced by Dex/VitD2 tDCs are functional and suppress proliferation of responder T cells.

### Dex/VitD2 tDCs used NF-κB, p38 MAPK and ERK1/2 to regulate their tolerogenic properties in the inflammatory environment

To decipher the molecular mechanisms that play a role in maintaining tolerogenic properties of tDCs, signaling pathways including p38 MAPK, c-Jun N-terminal kinases (JNK/SAPK), ERK1/2, NF-κB, indoleamine 2, 3 deoxygenase (IDO), mTOR, and STAT3, previously reported to affect DC maturation and orchestrate IL-10 and IL-12 production, were analyzed [9, 11, 13, 14, 22].

**Figure 4:**
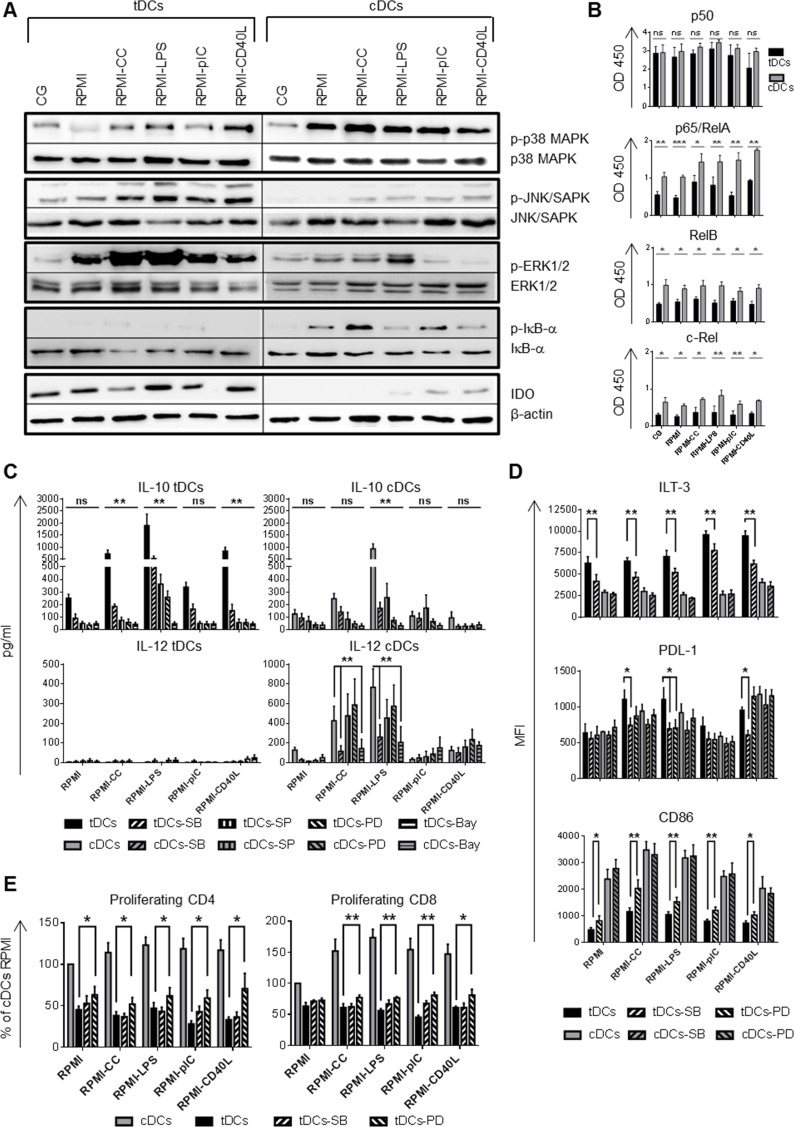
Different intracellular signaling pathways are triggered in tDCs and cDCs after mimicking *in vivo* DC activation DCs were differentiated in Cell Gro in presence (tDCs) or absence of Dex and VitD2 (cDCs) and activated with MPLA (CG). Then, tDCs and cDCs were washed, recultured in complete RPMI without tolerising factors and treated with following stimuli: cytokine cocktail (CC) described in Figure 1B or LPS (1 μg/ml) or polyI:C (25 μg/ml) or CD40L (1000 ng/ml) or they were left unstimulated (RPMI). **A.** After 60 min of restimulation, the phosphorylation of p38 MAPK, JNK/SAPK, ERK1/2, IκB-α and the level of IDO was analyzed by western blot analysis. Total p38 MAPK, JNK/SAPK, ERK1/2, IκB-α or β-actin in each sample were used as the equal loading control. One of three experiments performed is shown. **B.** After 90 min of restimulation, DNA-binding activity of NF-κB subunits was analyzed by colorimetric assay. **C.** Production of IL-10 and IL-12 after restimulation of tDCs and cDCs with CC, LPS, polyI:C and CD40L for 24 h in the presence of p38 MAPK inhibitor SB203580 (SB), JNK/SAPK inhibitor SP600125 (SP), ERK1/2 inhibitor PD98059 (PD), NF-κB inhibitor Bay 11-7082 (Bay) was evaluated by ELISA. **D.** ILT-3, PDL-1 and CD86 expression on tDCs and cDCs after restimulation with CC, LPS, polyI:C and CD40L for 24 h in the presence of p38 MAPK inhibitor SB203580 (SB) and ERK1/2 inhibitor PD98059 (PD) was evaluated by flow cytometry. **E.** Before restimulation, tDCs were pretreated with p38 MAPK inhibitor SB203580 (SB) and ERK1/2 inhibitor PD98059 (PD) and stimulated for 24 h. tDCs were then cocultered with allogeneic T cells. Proliferation was measured on day 6. Data represent mean ± SEM from at least three independent experiments. *p ≤ 0.05, **p ≤ 0.01, ***p ≤ 0.001 (paired *t*-test).

First, we checked whether MAPK, including p38 MAPK, JNK/SAPK and ERK1/2, are differentially regulated in tDCs and cDCs. As shown in Figure 4A, tDCs from Cell Gro expressed higher levels of activated JNK/SAPK, however, p38 MAPK and ERK1/2 were comparably activated in tDCs and cDCs. After re-exposing DCs to inflammatory stimuli, tDCs expressed higher level of activated JNK/SAPK, lower level of the activated p38 MAPK and markedly up-regulated ERK1/2 in contrast to cDCs.

Next, we found that tDCs expressed high level of immunoregulatory molecule IDO in all the stimulatory conditions tested. In contrast, IDO was absent or weakly expressed in cDCs. These results suggest that p38 MAPK, JNK/SAPK, ERK1/2 and IDO are differentially regulated in tDCs compared to cDCs, which might play a role in maintaining tolerogenic properties of tDCs after rechallenge.

Given that DC differentiation and maturation is associated with activation of NF-κB and Dex/VitD tDCs differentiation was shown to be mediated through the suppression of NF-κB pathway [6, 7], we tested whether LPS, polyI:C, CC or CD40L can reverse NF-κB suppression in the absence of tolerogenic factors. First, we documented that phosphorylation of IκB-α, a regulatory protein that inhibits NF-κB by complexing with and trapping it in the cytoplasm, was dramatically reduced in tDCs in all the stimulatory conditions tested. In contrast, IκB-α was phosphorylated in cDCs (Figure 4A). To quantify NF-κB activation, we analyzed DNA binding activity of NF-κB subunits p50, p65/RelA, RelB and c-Rel in the nucleus (Figure 4B). Dex/VitD2 tDCs from Cell Gro exhibited low binding activity of p65/RelA, and low binding activity of RelB, shown to reflect DCs maturation [23], and c-Rel, shown to be involved in IL-12 production [24], in nuclear extracts when compared to cDCs. DNA binding activities of p65/RelA, RelB and c-Rel in tDCs remained lower even after rechallenge in the absence of VitD2 and Dex. On the other hand, binding activity of NF-κB subunit p50, shown to create homodimers increasing production of IL-10 [25], was high in tDCs in all the conditions tested (Figure 4B).

Next, we determined how MAPK and NF-κB signaling pathway utilization contributes to inflammatory versus tolerogenic phenotype of DCs in response to LPS, CC, polyI:C and CD40L. Before stimulation, DCs were pretreated by p38 MAPK, JNK/SAPK, ERK1/2, and NF-κB inhibitor SB203580, SP600125, PD98059 and Bay 11-7082, respectively. Analyzing IL-10 and IL-12 production, we found that IL-10 production was significantly dependent on p38 MAPK, JNK/SAPK and ERK1/2 activation pathways after CC, LPS and CD40L restimulation in tDCs. Also Bay 11-7082 abrogated IL-10 production in tDCs. However, we observed the same situation only after LPS triggering in cDCs (Figure 4C). On the other hand, p38 MAPK and NF-κB inhibitor markedly down-regulated IL-12 production in cDCs after LPS and CC triggering, but did not affect IL-12 production in tDCs. Analyzing cell-surface molecules, we found that p38 MAPK inhibition down-regulated ILT-3 and PDL-1 expression in tDCs, in contrast to cDCs (Figure 4D). Moreover, ERK1/2 inhibitor down-regulated PDL-1 expression after LPS stimulation in tDCs but significantly up-regulated CD86 expression in tDCs in all the conditions tested (Figure 4D). Other inhibitors tested had no significant effect on ILT-3, PDL-1 and CD86 expression in tDCs (data not shown). The ability of p38 MAPK and ERK1/2 inhibitors to modulate IL-10 production and expression of costimulatory and inhibitory molecules in tDCs suggest an impact on subsequent T cell activation. By employing the allogeneic T cell activation model, we found that ERK1/2 inhibitor increased the ability of Dex/VitD tDCs to stimulate CD4+ as well as CD8+ T cell proliferation when compared to tDCs without ERK1/2 inhibitor (Figure 4E). Collectively, these data suggest the distinct pattern of activated signaling pathways in tDCs versus cDCs, with p38 MAPK, ERK1/2 and down-regulated NF-κB being important for maintaining down-regulated CD86 and up-regulated ILT-3 and PDL-1 expression, high IL-10 production and reduced allostimulatory potential of Dex/VitD tDCs.

### mTOR and STAT3 regulate IL-10 production and ILT-3, PDL-1 and CD86 expression in tDCs after restimulation

**Figure 5:**
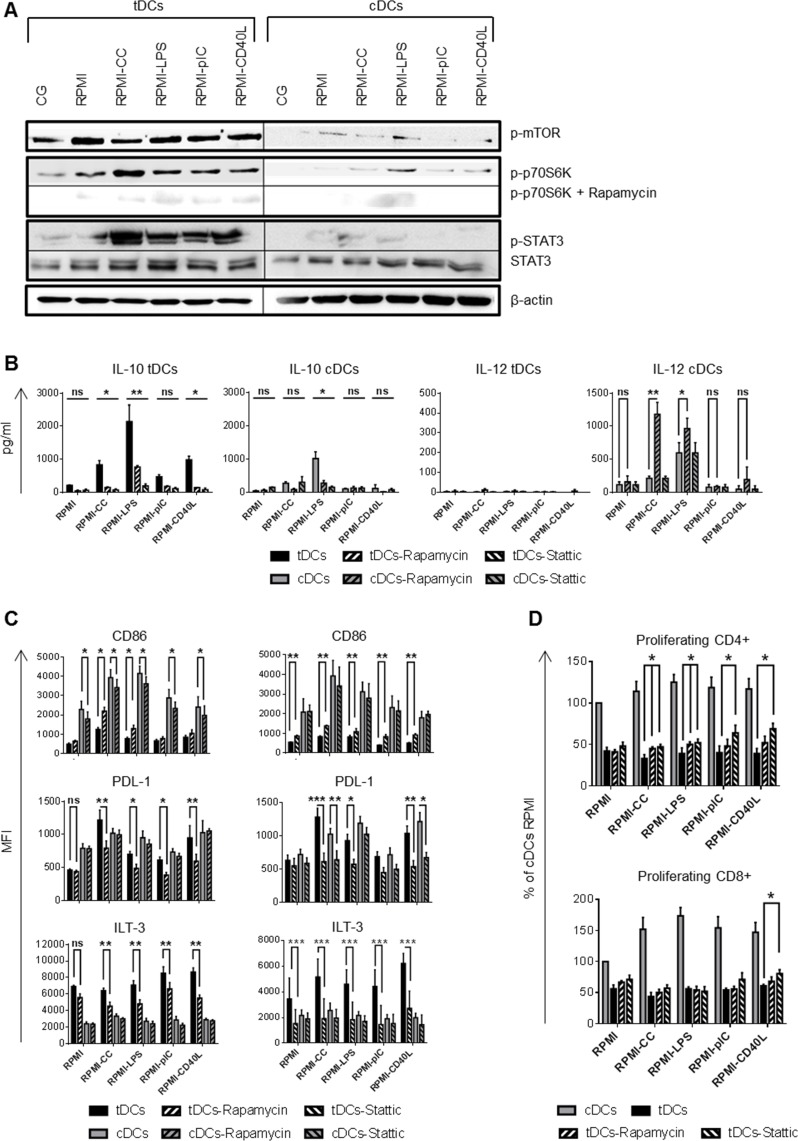
mTOR and STAT3 regulates tolerogenic properties of tDCs after restimulation DCs were differentiated in Cell Gro in presence (tDCs) or absence of Dex and VitD2 (cDCs) and activated with MPLA (CG). Then, tDCs and cDCs were washed, recultured in complete RPMI without tolerising factors and treated with cytokine cocktail (CC) described in Figure 1B or LPS (1 μg/ml) or polyI:C (25 μg/ml) or CD40L (1000 ng/ml) or they were left unstimulated (RPMI). When indicated, cells were pretreated with mTOR inhibitor rapamycin or STAT3 inhibitor Stattic for 30 min before restimulation. **A.** After 60 min of restimulation western blot analysis for phosphorylated mTOR, p70S6K and STAT3 were performed using specific mAbs. β-actin was used as the equal loading control. One of three experiments performed is shown. **B.** IL-10 and IL-12 production by DCs after 24 h of restimulation was measured by ELISA. **C.** Expression of CD86, PDL-1 and ILT-3 after restimulation with CC, LPS, polyI:C and CD40L in the presence of mTOR inhibitor rapamycin or STAT3 inhibitor Stattic for 24 h was evaluated by FACS analysis. **D.** Before restimulation, tDCs were pretreated with mTOR inhibitor rapamycin or STAT3 inhibitor Stattic and stimulated for 24 h. tDCs were then cocultered with allogeneic T cells. Proliferation was measured on day 6. Data represent mean ± SEM from at least 4 independent experiments. *p ≤ 0.05, **p ≤ 0.01, ***p ≤ 0.001 (paired *t*-test).

Recently, mTOR was found to coordinate pro- versus anti-inflammatory events in human monocytes and DCs by attenuating NF-κB and up-regulating STAT3 activity [11, 12]. Western blot analysis revealed that tDCs strongly phosphorylated mTOR and STAT3 after re-exposing to inflammatory stimuli while the phosphorylation of mTOR is weaker and phosphorylated STAT3 is barely detectable in cDCs. mTOR phosphorylation led to the phosphorylation of p70S6K, mTOR dependent event, that was abrogated by the mTOR specific inhibitor rapamycin (Figure 5A). To further corroborate the link between mTOR and STAT3 activation and IL-10 and IL-12 production as well as CD86, ILT-3 and PDL-1 expression, we performed blocking experiments of mTOR and STAT3 using chemical inhibitors rapamycin and Stattic, respectively. Upon mTOR and STAT3 inhibition tDCs reduced IL-10 production (Figure 5B). Rapamycin and Stattic down-regulated IL-10 production after LPS restimulation in cDCs (Figure 5B). However, in contrast to cDCs, where rapamycin treatment markedly increased IL-12 production after CC and LPS treatment, rapamycin was not able to restore IL-12 production in tDCs irrespective of the stimulatory agent. IL-12 production was unaffected after Stattic treatment in both DCs tested (Figure 5B). Furthermore, mTOR and STAT3 inhibition markedly reduced expression of tolerogenic markers PDL-1 and ILT-3 but significantly increased CD86 expression in tDCs after CC and LPS trigger (mTOR inhibition) or in all the conditions tested (STAT3 inhibition), respectively (Figure 5C). This was paralleled by a partial restoration of the ability of tDCs to stimulate especially CD4+ T cell proliferation when compared to tDCs cultivated without Rapamycin or Stattic (Figure 5D). Altogether, these data suggest that mTOR and STAT3 controls not only IL-10 production and ILT-3, PDL-1 and CD86 expression in tDCs after restimulation but also play a role in their immunoregulatory function.

### mTOR-dependent glycolysis regulate stable tolerogenic properties of tDCs after restimulation

TLR-induced proinflammatory maturation and activation of DCs was shown to be dependent upon PI3/Akt-mediated metabolic reprogramming, switching from oxidative phosphorylation (OXPHOS) to aerobic glycolysis [26]. mTOR is a downstream target of PI3/Akt and was shown to regulate glycolytic metabolism [27]. However, our data showed strong phosphorylation of mTOR in Dex/VitD tDCs after activation with TLR ligands, cytokine cocktail and CD40L which was not accompanied with tDCs maturation. In addition, mTOR inhibition down-regulated tolerogenic molecules ILT-3 and PDL-1 expression and IL-10 production in Dex/VitD tDCs. Therefore, we investigated whether mTOR activation in tDCs is accompanied with glycolytic activation and how glycolysis regulates stable tolerogenic profile of tDCs in the inflammatory environment.

**Figure 6:**
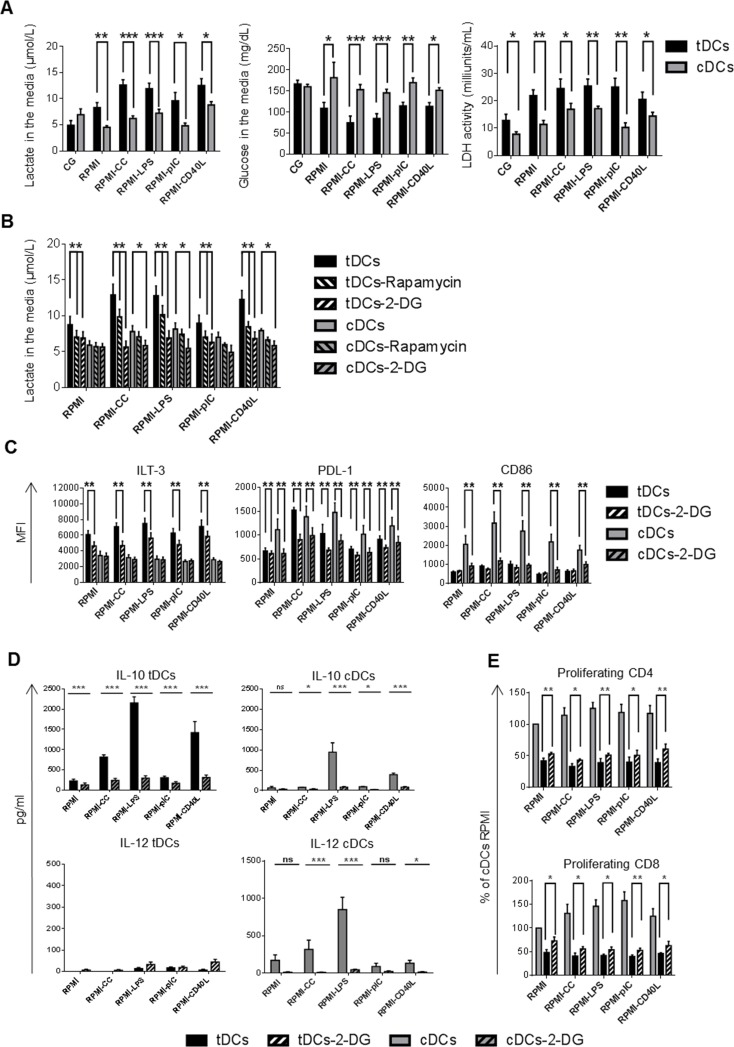
Enhanced glycolysis regulates tolerogenic phenotype and function of Dex/VitD tDCs DCs were differentiated in Cell Gro in presence (tDCs) or absence of Dex and VitD2 (cDCs) and activated with MPLA (CG). Then, tDCs and cDCs were washed, recultured in complete RPMI without tolerising factors and treated with following stimuli: cytokine cocktail (CC) described in Figure 1B or LPS (1 μg/ml) or polyI:C (25 μg/ml) or CD40L (1000 ng/ml) or they were left unstimulated (RPMI). When indicated, cells were pretreated with rapamycin or 10 mM 2-deoxyglucose (2-DG) for 30 min before restimulation. **A.** 24h later, supernatants were analyzed for the concentration of glucose and lactate as indicator of glycolytic activity. The activity of lactate dehydrogenase (LDH) was analyzed in cell lysates. **B.** 24 h later, suppression of glycolysis by treatment of DCs with rapamycin or 10 mM 2-deoxyglucose 30 min before restimulation was analyzed by evaluating the concentration of lactate in DC supernatants. **C.** ILT-3, PDL-1 and CD86 expression on DCs after 24 h of restimulation in the presence of glycolysis inhibitor 2-deoxyglucose was evaluated by FACS analysis. **D.** IL-10 and IL-12 production by DCs after 24h of restimulation was measured by ELISA. e Before restimulation, tDCs were pretreated with 2-DG and stimulated for 24 h. tDCs were then cocultered with allogeneic T cells. Proliferation was measured on day 6. Data represent mean ± SEM from at least 4 independent experiments. *p ≤ 0.05, **p ≤ 0.01, ***p ≤ 0.001 (paired *t*-test).

To investigate the glycolytic activity, glucose consumption and lactate production were analyzed in tDCs and cDCs supernatants as an indicator for glycolytic activity. As shown in Figure 6A, Dex/VitD tDCs cultured in Cell Gro secreted similar levels of lactate as cDCs. However, the restimulation of tDCs led to robust accumulation of lactate in cell supernatants that was accompanied with more pronounced gradual decrease in the media glucose content when compared to cDCs. Consistent with the increased glucose consumption and lactate production, tDCs after restimulation revealed higher activity of cellular lactate dehydrogenase, an oxidoreductase enzyme that catalyses the interconversion of pyruvate and lactate, in all the conditions tested compared to cDCs. These data suggest increased glycolysis in Dex/VitD2 tDCs in contrast to cDCs. To test whether mTOR regulates enhanced glycolytic metabolism in tDCs after restimulation, we performed blocking experiments using chemical mTOR inhibitor rapamycin. Rapamycin markedly down-regulated lactate generation in Dex/VitD tDCs (Figure 6B). Thus, restimulation of Dex/VitD tDCs is accompanied by enhanced glycolysis via mTOR activation pathway. Next, we tested whether enhanced glycolysis regulate tolerogenic properties of tDCs after restimulation by adding 10 mM 2-deoxyglucose (2-DG) which acts as an inhibitor of glycolysis and prevents generation of lactate to the DC cultures. Addition of 2-DG to the DCs cultures significantly prevented lactate generation in Dex/VitD tDCs (Figure 6B). Moreover, under these conditions, tDCs failed to up-regulate ILT-3 and PDL-1 molecules (Figure 6C) and markedly decreased IL-10 production (Figure 6D). Expression of CD86 as well as IL-12p70 production remained unaffected upon 2-DG treatment in tDCs in contrast to cDCs. On the other hand, inhibition of glycolysis in tDCs increased partially the ability to induce allogeneic CD4+ as well as CD8+ T cell proliferation (Figure 6E). Taken together, these data show that enhanced glycolysis alters expression of inhibitory molecules, IL-10 production and allostimulatory potential of Dex/VitD tDCs after mimicking *in vivo* subsequent pro-inflammatory activation.

## DISCUSSION

Our analysis showed that Dex/VitD tDCs maintain tolerogenic phenotype and function in the inflammatory environment in the absence of tolerogenic factors. We showed for the first time that stability of Dex/VitD tDCs in the inflammatory environment is orchestrated by down-regulated NF-κB, modest activation of p38 MAPK and strong activation of ERK1/2, mTOR and STAT3 molecules that regulate expression of CD86, ILT-3 and PDL-1, production of IL-10 and IL-12p70 and allostimulatory potential of Dex/VitD tDCs.

Recent studies showed a stable tolerogenic phenotype of Dex and/or VitD-treated DCs in terms of maturation markers expression and stable high IL-10 production upon repeated maturation with LPS or pro-inflammatory cytokines [20, 28, 29]. Our data showing stable low to intermediate CD86, CD83 and CD40 expression in contrast to high expression of ILT-3, TIM-3, TLR2 and PDL-1 after restimulation of tDCs corroborate and significantly extend recent findings about the stability of tDCs and indicate the preservation of anti-inflammatory phenotype of tDCs [3, 20]. ILT-3 signaling was shown to result in inhibition of NF-κB and p38 MAPK pathways in DC [30]. TLR-2, ILT-3 and PDL-1 signaling was reported to participate in Tregs induction [20, 31]. In our study, restimulation of tDCs, especially with CC and LPS, led to up-regulation of PDL-1 and ILT-3 expression and stable capacity to induce IL-10 producing CD4+ T cells possessing suppressive capacity. Therefore, our data predict that stable expression of TLR2, ILT-3 and up-regulation of PDL-1 after restimulation of tDCs might play a role in tolerance induction.

We showed that tolerogenic DCs restimulated by inflammatory signals maintained stable cytokine profile with high IL-10 production, up-regulated TGF-β, reduced TNF-α and virtually absent IL-12. The high production of IL-10 together with low production of pro-inflammatory cytokines TNF-α and IL-12 could favor Dex/VitD2 tDCs for immunotherapy.

Consistent with the observed tolerogenic phenotype, Dex/VitD2 tDCs restimulated by inflammatory signals not only showed a reduced ability to induce T cell proliferation, but also were capable of inducing T cells with low IFN-γ and high IL-10 production, by both CD4+ and CD8+ compartments when compared to T cell responses induced by cDCs. The reduction of IFN-γ positive T cells after restimulation with concomitant stable IL-10 positive T cells might be caused by switching T cell response rather toward Th2 due to higher IL-10 production from restimulated tDCs and cDCs [32].

As tDCs reported in this study produced TGF-β, which can induce Tregs as well as Th17 cells [33], we analyzed their Th17 polarizing activity by testing the production of IL-17 from T cells co-cultured with tDCs. We found that Dex/VitD2 tDCs significantly reduced IL-17A production from T cells, even after restimulation with pro-inflammatory stimuli, in contrast to cDCs. As Th17 as well as IFN-γ contributes for pathogenesis of autoimmune diseases [34], the reduction of T cells that secrete IL-17A and IFN-γ might halt or reverse harmful autoimmune processes in subjects with autoimmune disease. Importantly, the low production of IFN-γ and IL-17A with concomitant increased secretion of IL-10 was observed in CD4+ Tregs generated after repetitive stimulation with Dex/VitD2 tDCs and remained similar even upon restimulation with mature DCs. Therefore, cytokine alterations of T cells after priming with Dex/VitD2 DCs cannot be easily explained as the direct result of an insufficient stimulation.

Next, we focused on activation pathways triggered in Dex/VitD2 tDCs upon mimicking subsequent pro-inflammatory activation. We found stable down-regulation of NF-κB pathway in Dex/VitD2 tDCs, further documented by abrogated phosphorylation of IκB-α. In contrast to cDCs, Dex/VitD2 tDCs exhibited low nuclear translocation of NF-κB subunits p65/RelA, RelB and c-Rel that have been shown to up-regulate pro-inflammatory cytokine production [25]. Our data are consistent with the observation that extent of nuclear expression of RelB as a p50/RelB heterodimer in DCs correlates with the degree of maturation [23]. As c-Rel plays a role in IL-12 production [24], down-regulated levels of c-Rel in our tDCs reflect their abrogated ability to produce IL-12 even after secondary stimulation when the tolerogenic agents are absent. High levels of p50 in nucleus of tDCs can reflect the fact that p50 homodimers repress proinflammatory cytokine production but serve as transcriptional activators of IL-10 [25]. The link between high levels of p50 and high production of IL-10 in tDCs is supported by strong reduction of IL-10 production after treatment with NF-κB inhibitor Bay 11-7082 reported previously to block phosphorylation of p50 [35].

Our data support the use of distinct MAPK activation pathways in tDCs *vs* cDCs after restimulation with inflammatory stimuli. In tDCs, activation of p38 MAPK after restimulation is lower compared to cDCs. However, the experiments with p38 MAPK inhibitor show that p38 MAPK plays an important role in IL-10 production and expression of tolerogenic molecules ILT-3 and PDL-1 in tDCs. In contrast, p38 MAPK is markedly activated in cDCs after restimulation and controls mainly IL-12 production with no significant effect on the expression of tolerogenic molecules. We next show the significant ERK1/2 phosphorylation after restimulation with all stimuli tested in tDCs but only after LPS restimulation in cDCs. This might correlate with marked up-regulation of IL-10 production in these stimulatory conditions. Blocking experiments with ERK1/2 inhibitor PD98059 confirmed the role of ERK1/2 in IL-10 production after inflammatory trigger in tDCs and support the role of ERK1/2 activation in IL-10 secretion [36]. Moreover, blocking of ERK1/2 activation partially restored CD86 up-regulation, prevented PDL-1 up-regulation and partially restored allostimulatory potential of tDCs. These data suggest the distinct role of p38 MAPK and ERK in tolerogenic *vs* pro-inflammatory maturation. Corroborating our results, p38 MAPK and ERK were shown to regulate PDL-1 expression in different DCs types [37].

Dex/VitD2 tDCs also express high levels of IDO that remains stable after restimulation. As expression of IDO in tDCs and the ensuing production of tryptophan metabolites has been shown to induce direct suppression of effector T cell activity and concurrent expansion of Tregs [14, 15], stable IDO expression might support tolerogenic properties of Dex/VitD2 tDCs.

Finally, we newly documented that mTOR and STAT3 inhibition led to up-regulated CD86 expression, down-regulated ILT-3 and PDL-1 expression, down-regulated IL-10 production and increased ability to stimulate T cell proliferation in Dex/VitD2 tDCs after restimulation. This phenotype was not observed in control DCs in which surface expression of CD86 was down-regulated but PDL-1 and ILT-3 expression remained similar upon mTOR inhibition. Our data demonstrate the novel and important anti-inflammatory role of mTOR and STAT3 in Dex/VitD2 tDCs and brings additional knowledge about the versatile role of mTOR in DC activation. Recently, the PI3K/mTOR pathway has been documented as a negative regulator of TLR signaling in human monocytes and myeloid DCs. Rapamycin-treated myeloid immune cells display a strong Th1 and Th17 polarization [11] and are capable of blocking the anti-inflammatory effects of dexamethasone [38]. It might suggest that dexamethasone used for generation of our Dex/VitD2 tDCs requires active mTOR for maintaining its anti-inflammatory effects. On the other hand, mTOR was documented to be indispensable for monocyte-derived DC survival and differentiation [11, 12, 39]. Data from our work suggest that in Dex/VitD tDCs, mTOR pathway dictate the maintenance of tolerogenic DC phenotype.

Surprisingly, we showed that enhanced glycolysis modulated via mTOR signaling pathway regulate tolerogenic phenotype and function of Dex/VitD tDCs in the inflammatory environment by modulating CD86, ILT-3 and PDL-1 expression, IL-10/IL-12 ratio and ability to stimulate T cell proliferation. Our data are in a contrast to previous studies showing enhanced glycolysis and PI3/Akt/mTOR signaling pathway being indispensable for pro-inflammatory maturation and function of DCs and T cells [26, 27]. However, in line with our data, Ferreira et al. documented very recently that tolerogenic DCs generated by VitD3 use the activation of glucose metabolism controlled by the PI3/Akt/mTOR signaling pathway to promote tolerogenic phenotype and function [40].

Taken together, we report that our clinical grade Dex/VitD2 tDCs preserve their phenotypic and functional properties upon stimulation with a variety of biologically relevant inflammatory stimuli in the absence of tolerogenic factors. To our knowledge, this study describes for the first time the regulation of key activation pathways after restimulation of tDCs in the absence of tolerogenic agents. Our data show that tDCs employ distinct activation pathways such as p38 MAPK, ERK1/2, IDO, mTOR and STAT3 to maintain their tolerogenic phenotype and immunoregulatory function upon mimicking subsequent pro-inflammatory activation in contrast to cDCs characterized by strong activation of p38 MAPK and NF-κB. Distinct pattern of signaling pathways triggered by inflammatory stimuli can also serve as a feasible and robust identity test that would distinguish inflammatory and tolerogenic DCs in culture. This study on clinical grade tDCs provides a rationale for their testing in the clinical settings, such as in autoimmune diseases or transplantation.

## MATERIALS AND METHODS

### Reagents and Abs

Flow cytometry: commercial antibodies anti-CD86-FITC (clone 2231 FUN-1), CD274 (PD-L1)-FITC (clone MIH1), CD273 (PD-L2)-PE (clone MIH-18), HLA-DR-PE-Cy7 (clone L243), IFN-γ-FITC (clone 4SB3) were purchased from BD Biosciences; CD83-PerCP-Cy5.5 (clone HB15a) was purchased from Beckman Coulter; CD80-FITC (clone MAB104), CD40-PerCP-eFluor710 (clone 5C3), CD1a-PE-Cy7 (clone HI149) and CD4-PE-Cy7 (clone RPA-T4) were purchased from eBioscience; TLR2-FITC (clone T2.5), TIM-3-PE (clone F38-2E2), IL-10-PE (clone JES3-9D7), KI-67-PE (clone Ki-67) were purchased from BioLegend; CD14-PE-DL594 (clone MEM-15), CD11c-APC (clone BU15), CD3-AF700 (clone MEM-57), CD8-PE-Dy590 (clone MEM-31) were purchased from Exbio; CD85k (ILT-3)-PE (clone 293623), CD85d (IL-T4)-FITC (clone 287219) were purchased from R&D Systems. For western blot, anti-p-p38 MAPK, anti-p-ERK1/2, anti-p-JNK/SAPK, anti-p-IκB-α, anti-IDO, anti-p-mTOR, anti-p-STAT3, anti-p-p70S6K, anti-p38 MAPK, anti-ERK1/2, anti-JNK/SAPK and anti-STAT3 Ab were purchased from Cell Signaling Technology; anti-actin was from BioLegend.

### DC differentiation, stimulation and inhibition

Immature DCs were obtained from buffy coats of healthy donors as previously described [18]. Briefly, human peripheral blood mononuclear cells (PBMC) were isolated by Ficoll gradient and monocytes were separated by allowing 2 h of cell adhesion in 75-cm^2^ culture flasks (Nunc). DCs were generated by culturing monocytes for 6 days in GMP-grade Cell Gro DC medium (CellGenix) containing penicillin and streptomycin (100 U/ml and 100 μg/ml, respectively, Gibco) in the presence of GM-CSF (500 IU/ml, Gentaur) and IL-4 (20 ng/ml, CellGenix). Medium and cytokines were replenished on day 3. On day 6, DCs were harvested and seeded in 96-well plates (Nunc) at 1×10^6^cells/ml. On day 7, immature DCs were activated with vacci grade MPLA (2 μg/ml, Cayla-InvivoGen) for 24 hrs. To induce tDCs, DCs were treated with Dex on day 3 (1 μM, Medochemie) and Dex and VitD2-paricalcitol (1,5 ng/ml, Zemplar, Abbott Laboratories) on day 6. Control DCs (cDCs) were cultured without tolerising factors. For restimulation assays, tDCs and cDCs were washed and recultured in complete RPMI medium (Gibco) containing 5% human AB serum (Invitrogen) in the absence of tolerising factors for 24 h, with or without LPS (1 μg/ml, Sigma-Aldrich), polyI:C (25 μg/ml, Cayla-InvivoGen), megaCD40L™ (1000 ng/ml, Enzo Life Sciences) or mixture of pro-inflammatory cytokines containing IL-1β, TNF-α, IL-6 (all 10 ng/ml) and IFN-γ (100 ng/ml) (all from R&D systems). Signaling inhibitors were added 1 h before the start of experiments under the specified stimulation conditions. SB203580 (p38 MAPK inhibitor at 10 μM), SP600125 (JNK/SAPK inhibitor at 20 μM), PD98059 (ERK1/2 inhibitor at 20 μM), Bay 11-7082 (NF-κB inhibitor at 10 μM), Stattic (STAT3 inhibitor at 5 μM) and rapamycin (mTOR inhibitor at 100 nM) were obtained from Calbiochem and dissolved in dimethyl sulfoxide. Supernatants and cells were collected for further analysis.

### Flow cytometry

Cells (2×10^5^/well) were stained with fluorochrome-conjugated mAbs for 30 min at 4°C in PBS, washed and analysed on LSR Fortessa cell analyzer (BD Biosciences). Appropriate isotype controls were included. Data were analyzed using FlowJo software (Tree Star). DCs were gated according to the forward scatter, side scatter and CD11c+ parameters for analysis. Dead cells were excluded from the analysis based on DAPI (4′,6-diamidin-2-fenylindol) staining. For intracellular cytokine staining, T cells were stimulated with phorbol 12-myristate 13-acetate (PMA) (50 ng/ml, Sigma-Aldrich) plus ionomycin (1 μg/ml, Sigma-Aldrich) for 4-16 h in the presence of Brefeldin A (5 μg/ml, BioLegend) before analysing. After stimulation, cells were washed, incubated in Fixation/Permeabilization Buffer (eBioscience) for 30 min at 4°C, then washed in Permeabilization Buffer (eBioscience) and stained with appropriate monoclonal antibody (mAb) for 30 min at 4°C.

### DC cytokine production

Cell supernatants were harvested after 24 h of DC stimulation and frozen at −80°C until analysis. IL-10, IL-12p70, TNF-α and TGF-β concentrations were determined using Luminex assay (MILLIPLEX™ Human Cytokine/Chemokine Kit, Merck Millipore) and ELISA assay (DuoSet ELISA Kit, R&D systems) according to the manufacturer's instructions. Cell supernatants were acidified before measuring TGF-β levels according to the manufacturer's instructions.

### DCs and T cells cultures, allostimulatory assay

T cells were obtained from PBMC non-adherent fraction. tDCs or cDCs (2×10^4^) were cultured with allogeneic T cells (2×10^5^) in complete RPMI medium (Gibco) containing 5% human AB serum (Invitrogen). IL-2 (20 U/ml, PeproTech) was added on day 2, 5 and 7. For primary mixed lymphocyte reaction (MLR) assays, allogeneic T cells (2×10^5^) labelled with 5 μM carboxyfluorescein succinimidyl ester (CFSE) (Invitrogen) were incubated with tDCs or cDCs (2×10^4^). T cell proliferation was determined by the sequential dilution of CFSE fluorescence of T cells, as detected by flow cytometry on day 6. For detection of IFN-γ, IL-10 and IL-17A production by T cells, 2×10^4^ tDCs or cDCs were cultured with 2×10^5^ allogeneic T cells. Cytokine production was determined by intracellular staining by flow cytometry on day 6 (IFN-γ) and day 9 (IL-10). IL-17A production from cell culture supernatants was analyzed by ELISA on day 6.

### Expansion of regulatory T cells and suppression assay

Naïve CD4+ T cells (donor A) were purified by negative selection with The EasySep™ Human Naïve CD4+ T Cell Enrichment Kit (StemCell Technologies). Naïve CD4+ T cells were plated with allogeneic human leukocyte antigen (HLA)-mismatched Dex/VitD2 tDCs (donor B) in a 10:1 ratio for 6 d in complete RPMI (5% human AB serum) in a 24-well plate. IL-2 (20 U/ml, PeproTech) was added on day 2 and 5. Next, T cells were washed and rested for 2 d with complete RPMI (5% human AB serum) and IL-2 and subsequently restimulated with Dex/VitD2 tDCs under the same condition for 5 d. After 5 days, T cells were recovered and rested for 2 days before use in the suppression assay. T cells primed for two rounds with Dex/VitD2 tDCs are referred to as Tregs. CD4+ Tregs were tested for suppressive capacity in following MLR assay. CD4+ Tregs (donor A) were labeled with Vybrant DiD cell labeling solution (5 μM, Millipore), washed and plated in a round-bottom 96-well plate coated with 1:20 000 anti-CD3 mAb (clone MEM-57) with responder T cells (donor A) and MPLA-matured cDCs (not treated with Dex and VitD2) (donor B). cDCs were from the same donor as the Dex/VitD2 tDCs used to induce Tregs. Cells were plated in a Treg/Tresp/DCs ratio of 10:10:1 or 5:10:1. As additional controls, Tresp and Tregs were cultured alone or with cDCs. After 6 d, cells were recovered and proliferation of responder cells was analyzed by measuring Ki-67 by flow cytometry. Cell culture supernatants were recovered for IL-10, IFN-γ and IL-17A analysis.

### Western blot analysis

Cell lysates (2×10^6^ DCs) were prepared from cells cultured in Cell Gro or recultured in complete RPMI alone or with cytokine cocktail, LPS, polyI:C or CD40L for 1 h as previously described [18]. When indicated, rapamycin (100 nM) was added 1 h before stimulation. Cell lysates were subjected to 10% sodium dodecyl sulfate polyacrylamide gel electrophoresis and transferred to nitrocellulose membranes before being immunoblotted with indicated specific mAbs. The membranes were revealed by horseradish peroxidase-conjugated secondary Ab (Cell Signaling Technology) using the West Femto Maximum Sensitivity Substrate (Pierce). After stripping, the membranes were reprobed with an appropriate mAb as loading control.

### Preparation of nuclear extracts and colorimetric NF-κB assay

Nuclear extracts were prepared from DCs (2×10^6^) cultured in Cell Gro or recultured in complete RPMI alone or with cytokine cocktail, LPS, polyI:C or CD40L for 90 min using a nuclear extract kit (Active Motif). NF-κB DNA binding activity of p50, p65/RelA, c-Rel and RelB was measured as previously described [18].

### Metabolic quantification: lactate, glucose and lactate dehydrogenase measurements

Concentrations of lactate and glucose in DC culture supernatants were measured with Glycolysis Cell-based assay kit (Cayman Chemicals) and Glucose colorimetric assay kit (BioVision), respectively. When indicated, glycolysis was suppressed by treatment with 10 mM 2-deoxyglucose (Sigma) 1 h prior exposition of DCs cultured in Cell Gro into RPMI, LPS, CC, polyI:C or CD40L. LDH activity of the DCs extracts was measured with Lactate dehydrogenase activity assay kit (Sigma).

### Statistical analysis

Results were obtained from at least three independent experiments and are given as mean ± SEM. Two-tailed paired t-test was applied for data analysis using GraphPad PRISM 6. A value of p≤0.05 was considered statistically significant.

## SUPPLEMENTARY MATERIAL FIGURE


