# Supplementary material for: NF-κB, p38 MAPK, ERK1/2, mTOR, STAT3 and increased glycolysis regulate stability of paricalcitol/dexamethasone-generated tolerogenic dendritic cells in the inflammatory environment
Source: Oncotarget. 2015 May 22;6(16):14123–38. doi: 10.18632/oncotarget.4234 (PMC4546455; doi:10.18632/oncotarget.4234)
Supplement: Supplementary file 1 [file oncotarget-06-14123-s001.pdf]

NF-κB, p38 MAPK, ERK1/2, mTOR, STAT3 and increased glycolysis regulate stability of paricalcitol/dexamethasone-generated tolerogenic dendritic cells in the inflammatory environment

Supplementary Material

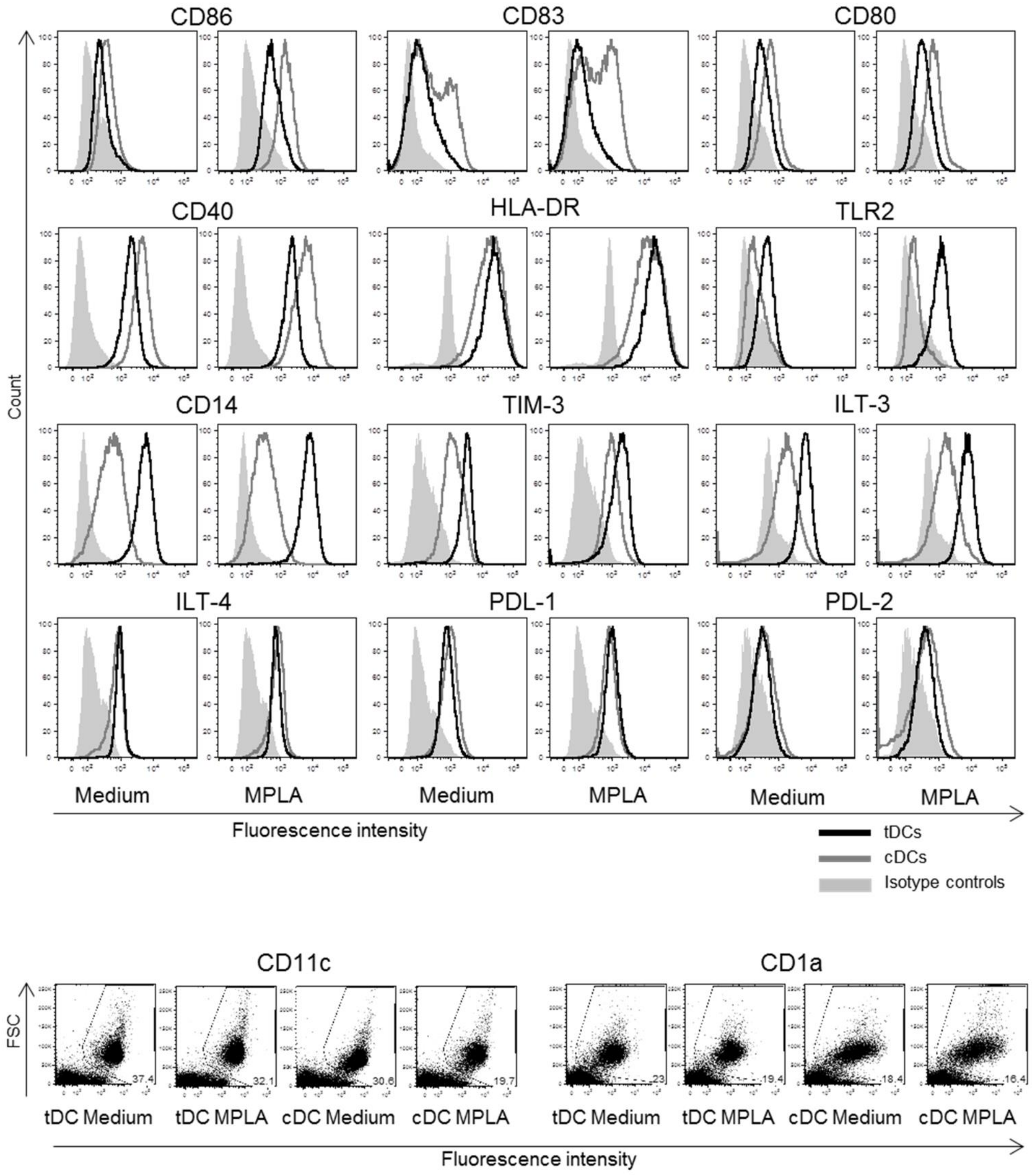

SUPPLEMENTAL FIGURE 1. Dex/VitD2 tDCs display tolerogenic features. DCs were differentiated in Cell Gro in presence (tDCs) or absence of Dex and VitD2 (cDCs) and then activated with MPLA. (**A**) Surface marker expression on tDCs (black lines) and cDCs (grey lines) before maturation (MEDIUM) and after final maturation with MPLA (MPLA) was evaluated by FACS. Light gray filled histograms represent isotype control mAb staining. (**B**) Representative dot plots of CD11c and CD1a positive cells are shown. Results are shown from representative experiments out of minimal 10 donors.
